# Effective Intense Pulsed Light Protocol in the Treatment of Moderate to Severe Acne Vulgaris

**DOI:** 10.3389/fmed.2022.946405

**Published:** 2022-07-01

**Authors:** Piccolo Domenico, Kostaki Dimitra, Crisman Giuliana, Dianzani Caterina, Avallone Gianluca, Giuffrida Roberta, Guarneri Fabrizio, Guida Stefania, Zalaudek Iris, Fusco Irene, Conforti Claudio

**Affiliations:** ^1^Skin Centers, Avezzano, Italy; ^2^Plastic and Reconstructive Surgery Department, Campus Biomedico University, Rome, Italy; ^3^Dermatology Clinic, Department of Medical Sciences, University of Turin, Turin, Italy; ^4^Department of Clinical and Experimental Medicine, Dermatology Section, University of Messina, Messina, Italy; ^5^Dermatology Unit, Department of Surgical, Medical, Dental and Morphological Science With Interest Transplant, Oncological and Regenerative Medicine, University of Modena and Reggio Emilia, Modena, Italy; ^6^Dermatology Clinic, Hospital Maggiore, University of Trieste, Trieste, Italy; ^7^Department of Pharmacology, University of Florence, Florence, Italy

**Keywords:** acne, lasers, IPL, papulo-pustulous acne vulgaris, young adults

Acne is defined as a chronic inflammatory-infectious disease of the pilosebaceous units, mainly affecting the face of young adults. Oral antibiotics, topical retinoids, azelaic acid, benzoyl peroxide, and isotretinoin represent the most common treatments, even though several adverse effects and a lack of durable remission, with a subsequent poor compliance by the patients, have been reported so far (1). On the other side, lasers have been proved to be effective and safe to treat acne; Intense Pulsed Light (IPL), particularly, demonstrates high efficacy rates, minimal discomfort, rapid recovery times, and excellent cosmetic and therapeutic outcomes (2).

We herein report our clinical experience with IPL for the treatment of moderate papulo-pustulous acne of the face. We used an IPL handpiece (Luxea Lazur handpiece, DEKA MELA srl, Calenzano, Italy) with the following parameters: wavelength 400 nm, fluence 8–9 J/cm^2^ and single-pulse mode of 30 ms duration. The protocol used was at least one session and at most 5 sessions separated by 2 weeks intervals.

The study included 62 patients (11 males and 51 females) with moderate to severe facial acne, not responsive to conventional therapies; patients suffering from mild acne were not enrolled in this study. The mean age was 20.95 ± 3.52 (minimum 18, maximum 39), with Fitzpatrick phototype I-II (*n* = 45, 72.58%), III (*n* = 13, 20.97%) and IV-VI (*n* = 4, 6.45%).

Majority of the patients had papulopustular acne (*n* = 50, 80.65%), whereas nodulocystic and comedonal acne were less common (*n* = 10, i.e., 16.13%, and *n* = 2, i.e., 3.23%, respectively).

Fifty-two (83.87%) patients were not on other anti-acne treatments. Concomitant medications were used in 10 patients (oral tetracycline in 6 cases, adapalene and benzoyl peroxide in 2 cases each).

According to the Hayashi score system (3), 20.97% (*n* = 13) of the participants had moderate acne, 48.39% (*n* = 30) severe and 30.65% (*n* = 19) very severe at baseline (Figure 1).

**Figure 1:**
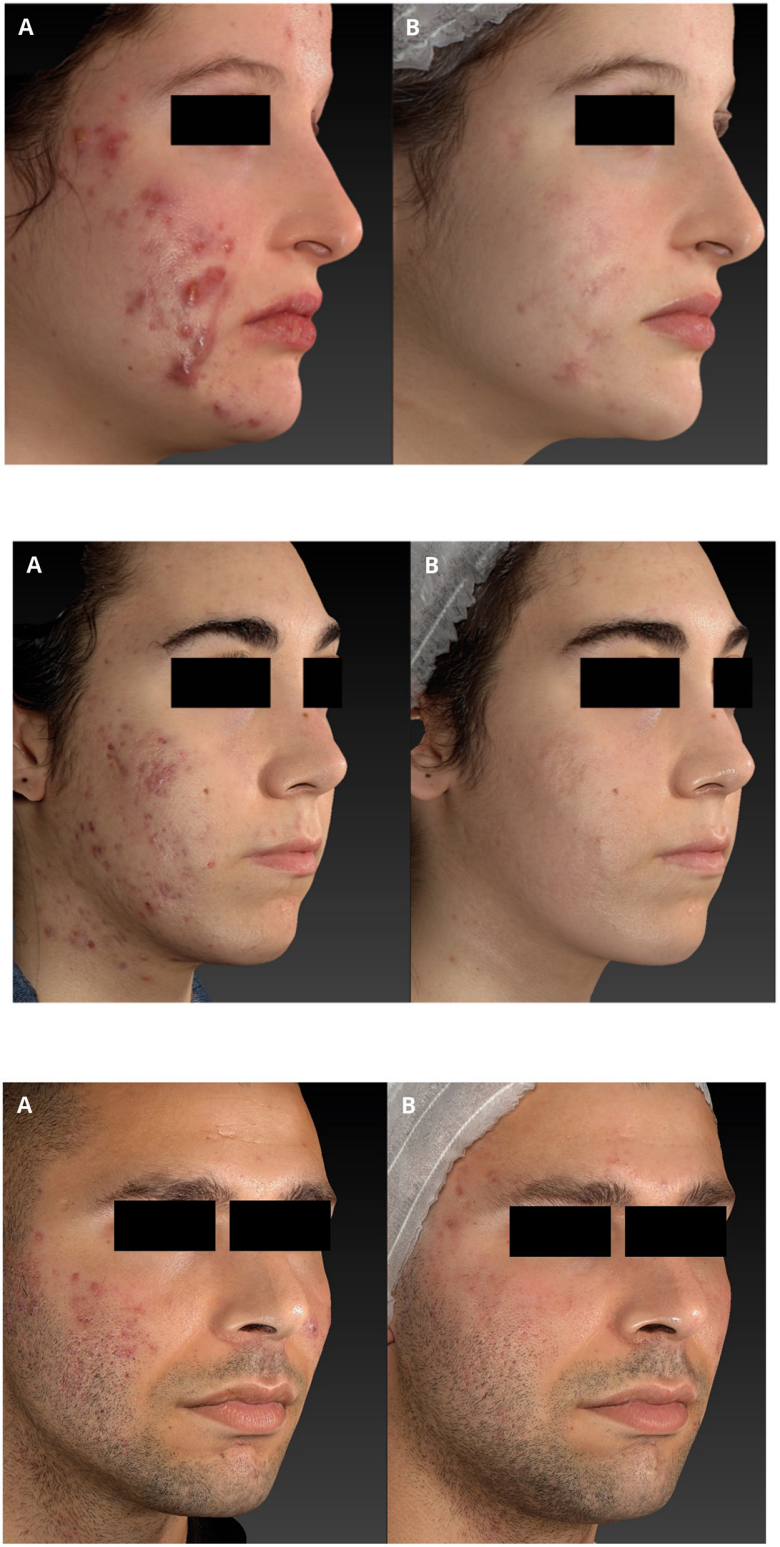
Nodulocystic acne before **(A)** and after **(B)** 4 sessions of IPL treatment (wavelength 400 nm) (top panel); Papulo-pustolous acne before **(A)** and after **(B)** 5 sessions of IPL treatment with notable improving of active acne lesions and acne scars (middle and bottom panel).

There was a significant (*p* = 2.84 × 10^−20^) improvement of Hayashi score after IPL at the final compared to before the treatment, with 48 patients (77.42%) with mild acne, 13 (20.97%) patients with moderate and only one (1.61%) with severe acne.

One patient (1.61%) received only one session of IPL, 37 (59.68%) two sessions, 17 (27.42%) three sessions, 5 (8.06%) four sessions and 2 (3.23%) five sessions.

No serious side effects occurring during or after the procedure were noted.

At the time of final assessment, 6 weeks after the last IPL session, the response to IPL treatment was considered excellent in 58.06% (*n* = 36) of patients, with complete regression of inflammatory lesions. Eighteen patients (29.03%) presented a good response, with few inflammatory lesions. Seven participants (11.29%) showed a moderate response to IPL, while only one (1.61%) presented a poor response.

There was no relationship between the response to treatment and any age, sex, phototype and type of acne lesions.

At 6 weeks follow-up, residual nodules were present in 13 patients (20.97%), brown macules in 3 (4.84%), papules and pustules in 13 patients (20.97%), papules, pustules and brown macules in 20 (32.26%) patients. No residual lesions were observed in 13 patients (20.97%).

Our study shows efficacy and safety of IPL in the treatment of acne vulgaris, as demonstrated by the statistically significant reduction of Hayashi score and by the absence of side effects; its validity is promising either as a complementary therapy during systemic or topical therapies (with the only exception of isotretinoin) or as a first therapeutic choice in patients with contraindications to normal therapies. The major limitations of this study are the limited number of the patients and concomitant medications in some of them. Future larger prospective studies are needed to evaluate the maintenance of long-term response.

## Author Contributions

PD, KD, CG, DC, AG, GR, GF, GS, ZI, and CC contributed to conception and design of the study. FI revised and submitted the manuscript. All authors contributed to manuscript revision, read, and approved the submitted version.

## Conflict of Interest

The authors declare that the research was conducted in the absence of any commercial or financial relationships that could be construed as a potential conflict of interest.

## Publisher's Note

All claims expressed in this article are solely those of the authors and do not necessarily represent those of their affiliated organizations, or those of the publisher, the editors and the reviewers. Any product that may be evaluated in this article, or claim that may be made by its manufacturer, is not guaranteed or endorsed by the publisher.
